# Mucous Disease or Chronic Intestinal Dyspepsia in Children

**Published:** 1905-02-25

**Authors:** 


### MUCOUS DISEASE OR CHRONIC INTESTINAL DYSPEPSIA IN CHILDREN.

A recent paper by Dr. J. Brunton Blaikie recalls the importance of the condition first described by Dr. Eustace Smith under the title of mucous disease. It is so common in childhood, so alarming in its manifestations, and so amenable to treatment, that it is surprising to find it not more generally recognised. The affection is commonest at the period of the second dentition, and for some years after its commencement. Its general symptoms, varying of course in severity with the duration and stage of the malady, are languor, pallor, loss of flesh, and an increased nervous irritability. The special symptoms are intestinal chiefly ; the belly becomes flabby, enlarged, and tympanitic, and the motions of the bowels irregular, periods of diarrhœa alternating with periods of constipation. This intestinal irregularity is associated with stools of a character always suggestive, and sometimes almost pathognomonic of the lesion. This character is given by the presence in them of a large excess of mucus, which gives the condition its name. The tongue is also peculiarly affected and presents a "glossy, slimy look . . . the aspect it would bear if brushed over with a solution of gum."

Such children rapidly become sallow, they complain of occasional abdominal pain, the skin becomes rough and harsh, and they are subject to occasional "bilious attacks." They are also almost constantly the subjects of an indifferent circulation, evidenced particularly by a perpetual coldness and clamminess of the feet. The alarming features of the condition above alluded to depend upon the close likeness of the symptoms to those presented by certain varieties of abdominal tuberculosis, and since the latter lesion is eminently grave while the former is eminently curable, the importance of a timely identification is obvious.

The diagnosis of mucous disease as opposed to abdominal tuberculosis rests chiefly upon the following features of the former disease. The sliminess of the stools, the absence of fever, the character of the tongue, the absence of physical signs of tuberculosis elsewhere in the body, the absence of abdominal tenderness and of the masses and doughiness which often mark the tuberculous lesions, and finally the uneven progress of the symptoms.

The treatment is mainly dietetic, the indication being to reduce to a minimum foods of the carbohydrate order, which are particularly prone to perversions in fermentation. Thus potatoes, peas, beans, and all root vegetables, fruit, cakes and pastry, should for a time be rigidly excluded, and bread permitted only in the most reduced quantities. The proper diet for these patients is one consisting almost entirely of good cow's milk, meat, eggs, and fish. At the same time certain methods of improving the bodily health, and especially the circulation, are of the highest importance. These are to be found in hydro-therapy. A hot bath in the morning, rapidly given, with water at a temperature of 105° F., followed by a douche with water at 75° F., and subsequent brisk rubbing with a rough towel, is of the utmost benefit, and should be continued for some months. If it is found that a healthy reaction of the skin is not readily induced, a glass of milk and an egg should be given half an hour before the bath, or, if necessary, the bath may be given at bed time instead of on rising. Bodily warmth being of high significance, great attention should be given to the clothing, the legs especially requiring protection, while a flannel binder round the abdomen serves a most useful purpose.

Of drugs, the best in Dr Smith's opinion is an alkaline mixture with a bitter. Thus bicarbonate of soda with infusion of calumba and 20 minims of tincture of myrrh. This author also recommends a drachm or two of compound decoction of aloes twice or three times a day after food. In these quantities this compound does not act as a purgative, and rapidly improves the digestive and nervous elements of the cond tion. Finally, it is well to give an occasional aperient, such as a drachm of compound liquorice powder, twice a week to remove accumulations of mucus, and under this treatment an uncomplicated case may be expected to recover completely in the course of two or three months.

